# Breastfeeding’s protective role in alleviating breast cancer burden: a comprehensive review

**DOI:** 10.1097/MS9.0000000000001914

**Published:** 2024-03-05

**Authors:** Emmanuel Ifeanyi Obeagu, Getrude Uzoma Obeagu

**Affiliations:** a Department of Medical Laboratory Science; b School of Nursing Science, Kampala International University, Kampala, Uganda

**Keywords:** breastfeeding, epidemiology duration of breastfeeding, lifelong impact, ovarian cancer, risk reduction, timing of breastfeeding

## Abstract

Breastfeeding, an essential aspect of infant care, has garnered recognition beyond its immediate health benefits, revealing a profound and lasting impact on women’s health. Emerging research has unveiled a compelling relationship between breastfeeding and its enduring role in reducing the risk of ovarian cancer. This narrative review aims to comprehensively examine the lifelong impact of breastfeeding on ovarian cancer prevention, transcending infancy and delving into the mechanisms and implications for women’s health. Epidemiological evidence consistently demonstrates an inverse association between breastfeeding and the risk of ovarian cancer. Prolonged durations of breastfeeding correlate with a significant reduction in the likelihood of developing ovarian malignancies, underscoring the protective influence of sustained lactation. The mechanisms underlying breastfeeding’s impact on ovarian cancer prevention involve hormonal modulation and cellular changes. Breastfeeding contributes to reduced ovulatory cycles and oestrogen exposure, mitigating hormonal influences linked to ovarian cancer development. Moreover, the cellular alterations induced by breastfeeding within the ovarian microenvironment create an environment less conducive to malignant transformations. In conclusion, this paper consolidates evidence demonstrating breastfeeding’s enduring impact on reducing ovarian cancer risk. It emphasizes the need for continued research, supportive interventions, and societal engagement to promote breastfeeding practices. Embracing breastfeeding not only provides immediate health benefits but also represents a formidable strategy in lifelong ovarian cancer prevention, offering a promising pathway towards enhanced women’s health and well-being.

## Introduction

Highlights

- Infancy.
- Breastfeeding.
- Epidemiological evidence and risk reduction.
- Mechanisms underlying breastfeeding’s influence on risk of ovarian cancer.
- Lifelong impact and critical time windows.
- Relationship of breast feeding and ovarian Cancer.
- Overcoming challenges and enhancing support.
- Implications for clinical and health policy-making.

Breastfeeding, a fundamental element of early infant care, has long been acknowledged for its multifaceted benefits to both infants and mothers. Beyond its well-established advantages in infant nutrition and immunity, recent scientific inquiry has shed light on an intriguing relationship between breastfeeding and its enduring impact on reducing the risk of ovarian cancer among women^1^. This paper seeks to explore comprehensively and elucidate the profound link between breastfeeding and ovarian cancer prevention, extending beyond infancy and illuminating the long-term protective effects of sustained lactation. Ovarian cancer remains a formidable challenge in women’s health, characterized by its late diagnosis and limited effective treatment options^2^. Against this backdrop, understanding preventive strategies becomes paramount. Breastfeeding has emerged as an intriguing area of study, showcasing promise in mitigating the risk of ovarian cancer among women^3^.

Recent epidemiological evidence has unveiled a compelling association between breastfeeding and a reduced risk of ovarian cancer. Numerous studies and meta-analyses have consistently demonstrated an inverse relationship, highlighting that longer durations of breastfeeding are significantly linked to a notable decrease in the likelihood of developing ovarian malignancies^4,5^. This review aims to delve into the compelling evidence supporting breastfeeding as a protective factor against ovarian cancer, emphasizing the cumulative and enduring impact of sustained lactation. Beyond the intuitive advantages of breastfeeding for infant health, investigations into the mechanisms behind breastfeeding’s influence on ovarian cancer prevention have revealed intriguing pathways. Hormonal modulation, specifically the reduction in ovulatory cycles and oestrogen exposure facilitated by breastfeeding, emerges as a pivotal factor^6^. Additionally, the cellular changes induced by breastfeeding within the ovarian microenvironment contribute to creating an environment less favourable for the initiation and progression of ovarian malignancies.

This paper seeks to unravel the lifelong impact of breastfeeding on ovarian cancer prevention, extending the discussion beyond the lactation period. By examining critical time windows and exploring optimal breastfeeding strategies, it aims to shed light on maximizing the protective effects against ovarian cancer throughout a woman’s life. However, despite the compelling evidence supporting breastfeeding’s role in ovarian cancer prevention, persistent challenges hinder widespread adoption and continuation of breastfeeding practices. Socio-cultural norms, inadequate support systems, and individual barriers pose significant obstacles. Addressing these challenges through comprehensive strategies involving societal engagement, education, workplace policies, and community initiatives is pivotal in fostering sustained breastfeeding practices for long-term ovarian cancer prevention. This paper consolidates the growing body of evidence highlighting breastfeeding’s enduring impact on reducing the risk of ovarian cancer. It underscores the imperative for continued research, supportive interventions, and societal engagement to harness the lifelong benefits of breastfeeding for women’s health and ovarian cancer prevention.

## Infancy

Infancy refers to the earliest stage of human development, generally spanning from birth to approximately two years of age. It represents a critical period characterized by rapid growth and development, during which a multitude of foundational milestones are achieved in various domains, including physical, cognitive, emotional, and social aspects^7^. This phase is marked by significant physiological changes and milestones in motor development, such as a baby’s first attempts at rolling over, sitting, crawling, and eventually walking. Infants also experience rapid brain development, laying the groundwork for various cognitive skills and sensory perceptions, which are crucial for their future learning and interactions with the world^8^.

Nutritionally, infancy is a period of high dependence on maternal or infant feeding, primarily through breastfeeding or formula feeding, providing the necessary nutrients crucial for growth and development. Breast milk, specifically tailored to meet an infant’s nutritional needs, is highly recommended due to its unique composition and numerous health benefits^9^.

Emotionally and socially, infants begin forming attachments to their caregivers and start developing basic communication skills. They respond to facial expressions, sounds, and gestures, gradually learning to express their needs and emotions, laying the groundwork for future social interactions and relationships. Infancy serves as a critical window for establishing the foundation of lifelong health and well-being. Positive early experiences and nurturing care during this period are fundamental for healthy brain development, emotional regulation, and building secure relationships, which can significantly impact a child’s future physical and mental health^10^.

Parental care, responsive interactions, and a nurturing environment play pivotal roles in shaping an infant’s growth and development during this formative stage. Early interventions and support systems aimed at ensuring optimal nutrition, healthcare, and stimulation are vital in promoting healthy development and laying the groundwork for a child’s future potential^11^. Understanding and catering to the unique needs of infants during this critical phase are essential for fostering healthy growth, maximizing developmental potential, and providing a solid foundation for a lifetime of well-being and success.

Ovarian cancer typically develops in the ovaries, which are part of the female reproductive system and play a crucial role in egg production and hormone regulation. The risk of ovarian cancer increases with age, and the majority of cases are diagnosed in postmenopausal women. While it is rare, there are some genetic conditions that can predispose individuals to certain types of cancer, including ovarian cancer. However, these conditions are not specific to infancy, and their impact on cancer risk becomes more apparent in adulthood. It’s crucial to focus on age-appropriate health considerations during infancy, such as routine vaccinations, proper nutrition, and overall well-being. If there are concerns about genetic predispositions in the family, it is advisable to consult with healthcare professionals or genetic counsellors who can provide personalized guidance based on the specific family history and genetic information^10,11^.

## Breastfeeding

Breastfeeding is a natural and fundamental process in which a mother feeds her infant or young child with breast milk produced by her mammary glands. It’s widely acknowledged as the optimal source of nutrition for newborns and infants due to its unique composition that fulfills the nutritional needs of the growing baby^12^. Breast milk provides a balanced combination of proteins, fats, carbohydrates, vitamins, and minerals, tailored to meet an infant’s changing nutritional requirements. It contains antibodies and immune-boosting components that help protect infants from infections and diseases, offering vital support to their developing immune systems^13^.

The benefits of breastfeeding extend beyond nutrition. It aids in the bonding process between a mother and her child, fostering emotional connections and providing comfort and security to the baby. Additionally, breastfeeding has been associated with numerous health advantages for both the infant and the mother^14^.

For infants, breastfeeding is linked to a lower risk of various infections, including respiratory and gastrointestinal illnesses. It also contributes to a reduced risk of developing chronic conditions later in life, such as obesity, diabetes, and certain allergies. Breastfeeding is believed to enhance cognitive development in children, potentially leading to improved intelligence and academic outcomes^15^. For mothers, breastfeeding aids in postpartum recovery by helping the uterus contract, reducing bleeding after childbirth. It also assists in burning extra calories, aiding in post-pregnancy weight loss. Breastfeeding has been associated with a decreased risk of certain cancers, such as breast and ovarian cancer. It may also help in reducing the risk of postpartum depression^16^.

The WHO recommends exclusive breastfeeding for the first six months of a baby’s life, followed by continued breastfeeding alongside complementary foods for up to two years or beyond^17^. Despite its numerous benefits, challenges to breastfeeding exist, including difficulties with latching, milk supply issues, societal stigma, and inadequate support systems for nursing mothers. Efforts to promote and support breastfeeding involve initiatives to educate mothers about its benefits, creating supportive environments in workplaces and public spaces, and providing access to lactation consultants and peer support groups. These measures aim to encourage and enable mothers to breastfeed successfully, ensuring the health and well-being of both mothers and their infants^18^.

## Epidemiological evidence and risk reduction

Epidemiological evidence consistently demonstrates a notable association between breastfeeding and a reduced risk of various health conditions, including ovarian cancer^19^. Research studies and meta-analyses have indicated a compelling inverse relationship between breastfeeding and the risk of developing ovarian cancer, highlighting the potential protective effect of breastfeeding against this particular malignancy^4,20^. Studies investigating the impact of breastfeeding on ovarian cancer risk have shown that longer durations of breastfeeding are correlated with a decreased likelihood of developing ovarian cancer^21,22^. The protective effect appears to strengthen with increased duration and exclusivity of breastfeeding, suggesting a dose-response relationship between breastfeeding and reduced risk of ovarian cancer.

The mechanisms underlying breastfeeding’s impact on ovarian cancer risk reduction are not entirely elucidated, but several factors are believed to contribute to this association. Hormonal influences play a significant role, as breastfeeding suppresses ovulation and decreases cumulative exposure to ovarian hormones such as oestrogen and progesterone, which may contribute to the development of ovarian cancer^23,24^. Moreover, the cellular changes induced by breastfeeding, including alterations in the ovarian microenvironment, potentially create conditions less conducive to the initiation and growth of malignant ovarian cells. These factors collectively contribute to the observed reduction in ovarian cancer risk among women who have breastfed^25^.

While epidemiological evidence consistently supports the notion of breastfeeding as a protective factor against ovarian cancer, it’s essential to consider other variables that may influence the association. Factors such as parity, age at first childbirth, genetic predisposition, and lifestyle factors can also impact ovarian cancer risk and may interact with breastfeeding in complex ways^26^. Epidemiological evidence strongly suggests that breastfeeding is associated with a decreased risk of ovarian cancer. Longer durations of breastfeeding seem to confer greater protection against this malignancy. Further research into the underlying mechanisms and potential interactions with other risk factors is warranted to better understand the intricate relationship between breastfeeding and ovarian cancer risk reduction.

## Mechanisms underlying breastfeeding’s influence on risk of ovarian cancer

The impact of breastfeeding on reducing the risk of ovarian cancer involves intricate biological mechanisms that affect hormonal regulation and cellular changes within the body. While the exact mechanisms are not fully understood, several factors have been suggested to contribute to the effect of breastfeeding on reducing the risk of ovarian cancer:

### Hormonal modulation

Breastfeeding affects hormonal levels in a woman’s body, specifically by suppressing ovulation and reducing the frequency of menstrual cycles. The hormonal changes associated with breastfeeding, such as lower levels of oestrogen and progesterone, contribute to a reduced cumulative lifetime exposure to these hormones. Elevated levels of oestrogen and progesterone have been linked to an increased risk of ovarian cancer, and by reducing their levels, breastfeeding may help mitigate this risk^27^. Breastfeeding associated with natural contraception through suppression of ovarian cycles may not only protect against the development of ovarian cancer but may also protect against the development of an abnormal ovarian teratoma that is presumed to occur spontaneously in ovarian cycles through fusion of the secondary oocyte with the polar body^28^.

### Proliferative effect on breast cells

Breastfeeding induces differentiation and maturation of mammary gland cells. This process leads to cellular changes that make breast cells less susceptible to malignant transformation. These changes may extend beyond breast tissue to other hormone-sensitive tissues, including the ovaries, potentially reducing the risk of developing ovarian cancer^27^.

### Immune system activation

Breast milk contains various immune-boosting factors that provide passive immunity to infants. The act of breastfeeding stimulates the mother’s immune system, enhancing her body’s ability to recognize and eliminate abnormal or cancerous cells. This immune-boosting effect may have implications for reducing the risk of ovarian cancer by bolstering the body’s natural defense mechanisms against malignant cell growth^29^.

### Inflammatory and angiogenic factors

Breastfeeding influences the production of inflammatory markers and angiogenic factors. Lower levels of certain inflammatory markers and angiogenesis-related proteins have been associated with breastfeeding, potentially creating an environment less conducive to tumour growth and progression in the ovaries^27^.

### Cellular microenvironment

Breastfeeding-induced changes in the breast tissue microenvironment might extend to other organs, including the ovaries. These alterations may affect the microenvironment in a way that makes it less hospitable for the initiation and growth of cancerous cells, contributing to reduced ovarian cancer risk^29^.

## Lifelong impact and critical time windows

The impact of breastfeeding on reducing the risk of ovarian cancer extends beyond the immediate postpartum period, potentially conferring lifelong benefits. Research suggests that the duration and timing of breastfeeding play pivotal roles in influencing the long-term protective effect against ovarian cancer, emphasizing critical time windows that maximize its impact^29^. Studies indicate that the duration of breastfeeding correlates with the degree of risk reduction for ovarian cancer. Prolonged durations of breastfeeding, particularly breastfeeding for a total of several months or years across multiple children, appear to yield more significant risk reduction. Each additional month of breastfeeding contributes to a cumulative reduction in ovarian cancer risk, highlighting the importance of sustained lactation^30,31^.

Initiation and continuation of breastfeeding immediately after childbirth are considered critical. Exclusive breastfeeding in the early months of an infant’s life may have a more pronounced impact on reducing ovarian cancer risk^31^. Consistency in breastfeeding practices across multiple births or lactation periods may amplify its protective effects against ovarian cancer in the long-term. The age at which a woman gives birth to her first child is also linked to ovarian cancer risk. Early age at first childbirth, particularly before the age of 30, coupled with subsequent breastfeeding, has been associated with a decreased risk of ovarian cancer. The combination of early childbirth and sustained breastfeeding may synergistically contribute to reduced ovarian cancer risk^32,33^.

**Figure 1:**
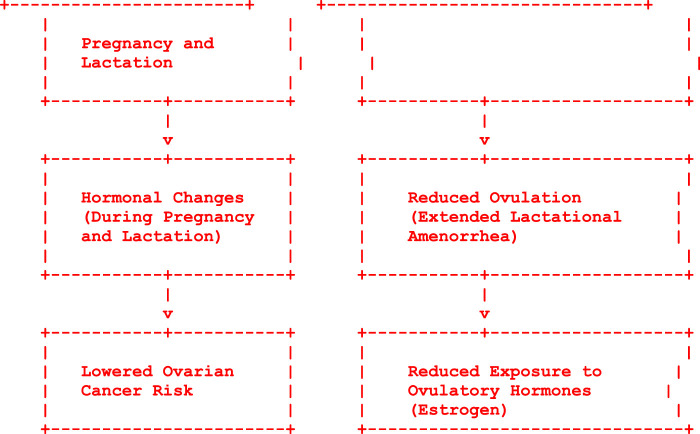
Flow diagram describing mechanism of prevention of ovarian cancer due to breast feeding.

Multiple childbirths combined with breastfeeding across these pregnancies may have a cumulative effect on reducing ovarian cancer risk. Women who have had multiple children and breastfed them for extended durations are observed to have a lower risk of ovarian cancer compared to nulliparous or less breastfeeding-experienced women^34^. Understanding these critical time windows and cumulative effects associated with breastfeeding practices can guide healthcare interventions and recommendations. Encouraging and supporting women to initiate breastfeeding early, breastfeed for extended durations, and maintain consistent breastfeeding practices across multiple pregnancies may potentially maximize the protective effects against ovarian cancer. While these associations are observed in epidemiological studies, additional research is warranted to further elucidate the specific impact of breastfeeding duration, timing, and consistency on lifelong ovarian cancer risk reduction. Identifying optimal breastfeeding strategies within critical time frames holds promise for enhancing women’s health and reducing the burden of ovarian cancer (Fig. 1).

## Relationship of breast feeding and ovarian cancer

Breastfeeding has been associated with a protective effect against ovarian cancer, meaning that women who have breastfed may have a lower risk of developing ovarian cancer compared to those who have not breastfed^21,35^. This relationship has been observed in various epidemiological studies, and several mechanisms are proposed to explain this protective effect. Breastfeeding can lead to hormonal changes in women, including a temporary suppression of ovulation. Prolactin, a hormone responsible for milk production, inhibits the release of gonadotropin-releasing hormone (GnRH), which, in turn, suppresses ovulation. This reduction in ovulation is believed to contribute to a lower risk of ovarian cancer, as repeated ovulation over a woman’s lifetime is considered a risk factor for the development of ovarian cancer^31,36^. Breastfeeding-induced amenorrhoea (temporary cessation of menstruation) can result in a reduced number of ovulatory cycles, and fewer ovulatory cycles have been associated with a decreased risk of ovarian cancer^27^. Breastfeeding is associated with lower levels of circulating oestrogen, which may contribute to the protective effect against ovarian cancer. High levels of oestrogen over a woman’s lifetime have been linked to an increased risk of ovarian cancer. Breastfeeding may help clear or prevent the development of premalignant cells in the breast and reproductive organs, potentially reducing the risk of cancer. The protective effect of breastfeeding appears to be dose-dependent, meaning that longer durations of breastfeeding and exclusive breastfeeding may offer greater protection^37^. Breastfeeding for as few as 3 months protects against EOC. Although this protection decreases over time, it persists for more than 30 years. Longer cumulative duration, increasing number of breastfeeding episodes, and earlier age at first breastfeeding episode increase protection^38^.

The process begins with pregnancy and lactation, which are associated with hormonal changes in a woman’s body. Pregnancy and lactation lead to alterations in hormonal levels, including increased levels of certain hormones and decreased exposure to ovulatory hormones like oestrogen. Lactation, especially exclusive breastfeeding, can result in extended periods of lactational amenorrhoea (absence of menstruation), reducing the frequency of ovulation. The decreased frequency of ovulation contributes to reduced exposure to ovulatory hormones, particularly oestrogen, which is known to be associated with the development of ovarian cancer. The cumulative effect of these factors results in a lowered risk of ovarian cancer associated with breastfeeding. Table 1: protective and risk factors of ovarian

**Table 1:** Protective and risk factors of ovarian cancer

Factors	Protective factors	Risk factors
Reproductive factors		
Age at first childbirth	Early age at first childbirth	Older age at first childbirth
No. pregnancies	Multiple pregnancies	Nulliparity (never having been pregnant)
Breastfeeding	Prolonged breastfeeding	Lack of breastfeeding
Oral contraceptive use	Long-term use of oral contraceptives	Never using oral contraceptives
Hormonal factors		
Hormone replacement therapy	Limited or no use of hormone replacement therapy (HRT)	Prolonged use of oestrogen-only HRT
Menopausal hormone therapy	Limited or no use of menopausal hormone therapy	Use of oestrogen-only menopausal hormone therapy
Genetic factors		
Family history	No family history of ovarian cancer	Family history of ovarian or breast cancer
Genetic mutations	Absence of specific genetic mutations (e.g., BRCA1, BRCA2)	Presence of specific genetic mutations (e.g. BRCA1, BRCA2)
Lifestyle factors		
Healthy diet	Balanced and healthy diet	High-fat diet, low in fruits and vegetables
Physical activity	Regular physical activity	Sedentary lifestyle
Environmental factors		
Occupational exposures	Limited exposure to certain occupational hazards	Prolonged exposure to certain occupational hazards
Medical history		
Gynaecological surgery	Protective effect of tubal ligation and hysterectomy	Ovarian surgery (may increase risk)
Other factors		
Inflammation	Anti-inflammatory lifestyle and diet	Chronic inflammation
Obesity	Healthy weight and BMI	Obesity

### Overcoming challenges and enhancing support

Overcoming challenges and enhancing support systems are crucial in fostering and sustaining breastfeeding practices, which play a pivotal role in reducing the risk of ovarian cancer^38^. Promoting awareness about the benefits of breastfeeding, including its role in reducing ovarian cancer risk, is fundamental^26^. Educating healthcare providers, expectant mothers, families, and communities about the importance of breastfeeding and its long-term health implications can encourage more informed decisions and garner support for breastfeeding. Implementing breastfeeding-friendly workplace policies is crucial for employed mothers. Providing adequate maternity leave, flexible work hours, designated lactation rooms, and breastfeeding breaks can facilitate continued breastfeeding after returning to work, removing barriers that might otherwise impede breastfeeding continuation^39,40^.

Access to skilled lactation consultants, breastfeeding support groups, and professional guidance can significantly impact breastfeeding success. Healthcare providers equipped with breastfeeding knowledge can offer guidance, address concerns, and provide support to mothers during their breastfeeding journey. Establishing community-based support groups, peer counselling programs, and breastfeeding networks can create a supportive environment for nursing mothers. Peer support from other breastfeeding mothers offers encouragement, shared experiences, and practical advice, fostering confidence and perseverance in breastfeeding^41^. Challenging societal stigmas surrounding breastfeeding in public spaces is essential^42^. Initiatives to normalize breastfeeding, reduce stigma, and create a culture that supports breastfeeding in public can promote greater societal acceptance and encouragement for breastfeeding mothers.

Encouraging involvement and support from partners, family members, and friends is crucial. A supportive social network that values and encourages breastfeeding can alleviate stress and provide invaluable emotional support to breastfeeding mothers^41^. Providing easily accessible and culturally sensitive breastfeeding resources, information, and educational materials in multiple languages can empower diverse communities and ensure that all mothers have access to vital breastfeeding support. By implementing these strategies, communities, healthcare systems, and policymakers can create an environment that fosters and supports breastfeeding practices. Overcoming barriers and enhancing support structures for breastfeeding not only benefits individual mothers and infants but also contributes to reducing the risk of ovarian cancer and promoting overall women’s health and well-being^41^.

## Implications for clinical and health policy-making

The implications of breastfeeding’s impact on reducing the risk of ovarian cancer have far-reaching implications for clinical practice and health policy-making. Understanding and acknowledging the association between breastfeeding and ovarian cancer risk reduction can influence clinical guidelines, healthcare practices, and policy decisions in several ways:

### Clinical guidelines and healthcare practices

Healthcare providers play a pivotal role in promoting and supporting breastfeeding^42^. Incorporating information about breastfeeding’s protective effects against ovarian cancer into clinical guidelines and healthcare practices can encourage healthcare providers to educate and counsel women about the long-term health benefits of breastfeeding. Providing evidence-based information during prenatal care, childbirth, and postpartum visits can empower women to make informed decisions regarding breastfeeding.

### Targeted interventions and support

Recognizing breastfeeding as a potential preventive measure against ovarian cancer can drive targeted interventions and support programs^43^. Tailoring healthcare interventions to promote breastfeeding practices, particularly among populations with higher ovarian cancer risks or lower breastfeeding rates, can help reduce disparities and improve health outcomes.

### Public health policies and initiatives

Integrating breastfeeding support into public health policies and initiatives is essential. Implementing policies that support breastfeeding-friendly environments in workplaces, public spaces, and healthcare facilities can facilitate sustained breastfeeding practices. Promoting breastfeeding as a public health priority can have broader implications for reducing the overall burden of ovarian cancer and improving women’s health outcomes.

### Investment in research and education

Continued investment in research focused on breastfeeding’s impact on ovarian cancer prevention is imperative. Further research can elucidate the mechanisms underlying this association, explore optimal breastfeeding strategies, and identify high-risk populations. Additionally, educational initiatives aimed at healthcare professionals, policymakers, and the general public can raise awareness and advocate for breastfeeding-friendly policies and practices.

### Health equity and access

Addressing disparities in breastfeeding rates among different socioeconomic, cultural, and geographic groups is essential^44,45^. Health policies that prioritize equitable access to breastfeeding support, resources, and education can help bridge gaps and ensure that all women have access to the benefits of breastfeeding, thereby contributing to reducing ovarian cancer risk across diverse populations.

## Conclusion

The intricate relationship between breastfeeding and the reduction of ovarian cancer risk illuminates a profound intersection between early maternal-child health practices and long-term women’s health outcomes. Extensive epidemiological evidence underscores the significant association between breastfeeding and a decreased likelihood of developing ovarian cancer, highlighting the multifaceted impact of sustained lactation. Embracing and supporting breastfeeding practices not only benefits infant health but also presents a formidable strategy in ovarian cancer prevention and women’s health promotion. Continued research, advocacy, and comprehensive support structures are pivotal in harnessing the lifelong benefits of breastfeeding, offering a promising pathway towards enhancing women’s health and well-being on a global scale. (Fig. 1).

## Ethical approval

Not applicable as this a review.

## Consent

Not applicable as this a review.

## Source of funding

No funding was received for writing this review paper.

## Author contribution

E.I.O. performed the following roles: conceptualisation, methodology, supervision, draft witing, editing and approval before submission.

## Conflicts of interest disclosure

The author declares no conflict of interest.

## Research registration unique identifying number (UIN)

Not applicable as this a review.

## Guarantor

Not applicable as this a review. It does not have any data.

## Data availability statement

Not applicable as this a review.

## Provenance and peer review

It is not invited.
